# Environmental sustainability from anesthesia providers’ perspective: a qualitative study

**DOI:** 10.1186/s12871-023-02344-1

**Published:** 2023-11-17

**Authors:** Greta Gasciauskaite, Justyna Lunkiewicz, Donat R. Spahn, Corinna Von Deschwanden, Christoph B. Nöthiger, David W. Tscholl

**Affiliations:** Institute of Anesthesiology, University and University Hospital Zurich, Raemistrasse 100, Zurich, 8091 Switzerland

**Keywords:** Global health, Climate change, Environmental sustainability, Carbon footprint, Anesthesiology, Greenhouse gases, Ozone depletion, Waste management.

## Abstract

**Background:**

The world faces a significant global health threat – climate change, which makes creating more environmentally sustainable healthcare systems necessary. As a resource-intensive specialty, anesthesiology contributes to a substantial fraction of healthcare’s environmental impact. This alarming situation invites us to reconsider the ecological health determinants and calls us to action.

**Methods:**

We conducted a single-center qualitative study involving an online survey to explore the environmental sustainability from anesthesia providers’ perspectives in a center implementing internal environmentally-sustainable anesthesia guidelines. We asked care providers how they perceive the importance of environmental issues in their work; the adverse effects they see on ecological sustainability in anesthesia practice; what measures they take to make anesthesia more environmentally friendly; what barriers they face in trying to do so; and why they are unable to adopt ecologically friendly practices in some instances. Using a thematic analysis approach, we identified dominating themes in participants’ responses.

**Results:**

A total of 62 anesthesia providers completed the online survey. 89% of the participants stated that environmental sustainability is essential in their work, and 95% reported that they implement measures to make their practice greener. A conscious choice of anesthetics was identified as the most common step the respondents take to reduce the environmental impact of anesthesia. Waste production and improper waste management was the most frequently mentioned anesthesia-associated threat to the environment. Lacking knowledge/teaching in sustainability themes was recognized as a crucial barrier to achieving ecology goals.

**Conclusions:**

Sustainable anesthesia initiatives have the potential to both encourage engagement among anesthesia providers and raise awareness of this global issue. These findings inspire opportunities for action in sustainable anesthesia and broaden the capacity to decrease the climate impact of health care.

**Supplementary Information:**

The online version contains supplementary material available at 10.1186/s12871-023-02344-1.

## Background

Climate change is defined as the world’s greatest global health challenge of the 21st century [1]. International organizations such as the Intergovernmental Panel on Climate Change call for fundamental and transformative change at every level of our personal and professional lives [2]. Global warming affects human life and health in many ways: the essential elements of healthy living – drinking water, nutritious food, clean air and secure shelter – are under threat. The healthcare sector significantly contributes to the climate crisis, accounting for over 4% of global CO2 emissions [3, 4]. Furthermore, healthcare practices lead to smog formation, acidification, the release of carcinogenic and non-carcinogenic air toxins, and waste production [5]. The situation escalated during the COVID-19 pandemic: the use of disposable items increased, with nearly 65 billion gloves and 129 billion masks discarded worldwide each month [6]. At the United Nations Climate Change Conference in Glasgow, United Kingdom (COP26) in 2021, 50 countries declared their commitment to low-carbon, sustainable health systems, with 14 countries aiming for net-zero health by 2050, and more countries have signed on since the conference [7]. As a highly technical, resource-intensive discipline, anesthesia practice accounts for a significant portion of healthcare’s CO2 emissions [8–10]. With growing calls to address the significant role of anesthesia practice in exacerbating climate change, volatile anesthetics have received increased attention, primarily due to their potent greenhouse gas properties. These volatile anesthetics undergo minimal in vivo metabolism and are released into the troposphere with minimal changes, accounting for over 95% of their emissions [11]. In particular, sevoflurane and desflurane persist in the troposphere for approximately 1.1 and 14 years, respectively [12]. Inhaled anesthetics can account for 50% of perioperative emissions [13] and 5% of hospital emissions [14]. Additionally, 30% of daily medical waste is produced in operating rooms; anesthesia practice is responsible for approximately 25% of it, of which 40% is potentially recyclable [15]. In recent years, numerous anesthesiology societies have published recommendations on how anesthesiologists can contribute to a reduction of the CO2 footprint [16–18]. The World Federation of Societies of Anesthesiologists has outlined core principles to guide anesthesia providers in the transition to environmentally sustainable practice, including choosing medications and equipment; minimizing waste and overuse of resources; and addressing environmental sustainability in education, research, quality improvement, and leadership activities [19].

Although interest in environmental sustainability in anesthesia practice is growing, implementing sustainable practices still needs to overcome many barriers. Several studies have shown that only about a third of anesthesiologists incorporate ecological practices into their daily work [20–22], which motivates the initiation of a systematic investigation of the obstacles and facilitators to sustainable anesthesia practice. This is a cause for concern, which invites us to reflect on how to systematically raise awareness and implement environmental sustainability in everyday work practice.

Climate change could undermine the progress made in global health for decades [23, 24]. However, this alarming situation gives us a powerful opportunity to redefine the environmental and social health determinants [1]. To shed light on the current condition of environmentally sustainable practices, attitudes, and knowledge among anesthesiologists, we conducted an online survey regarding environmental sustainability. Considering that healthcare professionals are leaders having an opportunity to influence changes at the local- and global levels, it is crucial to better understand their opinions and needs concerning the topic.

## Methods

### Approval and consent

The study protocol was reviewed by the Cantonal Ethics Committee of the Canton of Zurich, Switzerland, which issued a declaration of no objection (Req-2023-00358) and waived the need for ethical approval for the current study. The participants’ consent to participate was implied by their completion of the questionnaire and its submission, as approved by the Clinical Trials Center of the University Hospital Zurich. In this way, the informed consent was obtained from all the participants. Participation was voluntary and without financial compensation.

### Study design

We, a scientific team of the Anesthesia Department of the study center, conducted a single-center qualitative descriptive study to investigate anesthesia providers’ perspectives regarding environmental sustainability in anesthesia. The study was conducted at the University Hospital Zurich, Institute of Anesthesiology, Switzerland, over two consecutive weeks in March and April 2023. We report the study using the Strengthening the Reporting of Observational Studies in Epidemiology (STROBE) checklist for cross-sectional studies [25] and Standards for Reporting Qualitative Research (SRQR) checklist [26].

### Environmental sustainability initiatives within the study center

The Study Center is active in many ways to strengthen environmental sustainability.

In 2021–2022, several changes were introduced at the Institute of Anesthesiology to make anesthesia management more environmentally friendly. In December 2021, desflurane was eliminated, leaving it only as a reserve agent for patients undergoing prenatal myelomeningocele repair surgery. This initiative was followed by other ecological anesthesia management promoting changes provided in Table 1.

**Table 1:** Enforced package of measures to promote green anesthesia at the study center

Change implemented at the study center to promote greener anesthesia practice	Comments
Desflurane is no longer used	Desflurane is retained only as a reserve agent for patients undergoing prenatal myelomeningocele repair surgery.
Nitrous oxide, N_2_O, is no longer used	-
TIVA^*^ with propofol is the standard method of anesthesia unless medically indicated differently	• 1% propofol for procedures < 1 h; • 2% for procedures > 60 min.
The indications for inhalational anesthesia are limited	• Polytoxicomania; • Opioid tolerance (e.g. long-term ICU^**^ patients, chronic pain patients); • Severe sepsis and burns with high volume of distribution; • Severe bronchial obstruction; • Cardiac ischemia; • Adults with needle phobia for awake inhalation induction; • Propofol intolerance; • Fetal surgical procedures; • Ophthalmologic procedures; • Pediatric anesthesia.
Unnecessarily high intraoperative FiO2^***^ values should be avoided	Respirator settings after intubation: FiO2^***^ ≤0.4, aiming to maintain normoxia (SpO2^****^ ≥94%).
Unnecessarily high fresh gas flow should be avoided	Usually with ≤ 1 L/min after reaching the steady state.

*TIVA – total intravenous anesthesia

**ICU – intensive care unit

***FiO2 – fraction of inspired oxygen

****SpO2 – oxygen saturation

### Online questionnaire

We created an online survey using Google Forms (Alphabet Inc., Mountain View, CA, USA), which we e-mailed to all the anesthesia care providers at the study center, including staff anesthesiologists, residents and nurses. The questionnaire was initially sent on March 23, 2023; one week later the same participants received a reminder. The information collection was completed on April 6, 2023, when thematic saturation was reached.

In the questionnaire invitation, we informed participants that the survey takes approximately 8 min to complete and that participation is voluntary. The translated survey invitation is provided in Appendix 1.

The survey consisted of ten questions: four open-ended, requiring free text comments, and six closed-ended (Appendix 2). The first five questions focused on how important environmental sustainability is to respondents personally in their work; what negative ecological impacts they identify in their professional practice; whether they are taking steps to make their work more environmentally friendly; what barriers they face when trying to adopt ecological practices in their professional routine; and what are the reasons for the occasional non-compliance with established internal environmental sustainable anesthesia guidelines.

The last five questions assessed the demographics of the participants. This included age, sex, position (staff anesthesiologist, resident, nurse), and the number of years survey respondents had practiced anesthesia. The last question concerned whether the participants started working at the study center before or after implementing environmentally sustainable changes in anesthesia management.

We developed a comprehensive study design to address potential sources of bias from the outset. To minimize non-response bias [27], we made the questionnaire clear and concise and sent a follow-up reminder to increase participation. To reduce the effect of self-selection bias [28], we held several presentations on the impact of anesthesia on climate change to raise awareness and interest in the topic. To reduce information bias [29], we designed the survey instrument based on the following steps to create a valid and reliable instrument. We began by defining the objectives of the survey. A comprehensive literature review informed the structure of our questionnaire, drawing on relevant studies in health care settings [3, 20–22, 30]. Expert input was sought to assess content validity, and a pilot study with a target group of three professional colleagues helped to refine question wording and options.

### Data analysis

#### Open-ended questions

Collected responses to open-ended questions were translated from German to English using an online translator DeepL (DeepL GmbH, Cologne, Germany). The complete translated answers are provided in Appendix 3.

Using a thematic analysis six-phase approach [31], we identified the themes that dominated participants’ responses in each open question separately. After completing the data collection phase and familiarizing with the data, the research team discussed the overall impressions from the collected answers and generated ideas for potential codes. First, two team members, GG and JL, independently analyzed the interview responses; then, they discussed the consensus regarding the codes of the ideas shared by the participants. The generation of clear definitions and names for each theme and the final decisions were made in a joint discussion.

#### Closed-ended questions

The closed-ended responses analysis and figures were made using Microsoft Word and Excel (Microsoft Corp., Redmond, WA, USA). Demographic data are presented as numbers and their percentage distribution or as median and interquartile ranges.

## Results

### Participant characteristics

Of 343 anesthesia team members contacted via email, 62 (18.1%) completed an online survey. Staff anesthesiologists, residents and nurses accounted for approximately equal proportions. The least experienced anesthesia team member had less than one year of practice, and the most experienced – 35 years. More than three-quarters of the participants started working at the study center before the sustainable changes at the study center were implemented.

Detailed information on the study participants is provided in Table 2.

**Table 2:** Participant characteristics

Participants (n = 62)	
Sex	
Female	30 (48.4%)
Male	31 (50%)
Other gender identity	1 (1.6%)
Participant age in years, median (IQR)	37 (33–37)
Work in anesthesia experience in years, median (IQR)	8 (4–11)
Role	
Nurse anesthetist in training	1 (1.6%)
Certified nurse anesthetist	17 (27.4%)
Resident 1–2 years of training	7 (11.3%)
Resident 3–5 years of training	11 (17.7%)
Resident with > 5 years of experience	5 (8.1%)
Staff anesthesiologist	20 (32.3%)
Senior consultant	1 (1.6%)
Start of the work at the study center	
Before implementing environmentally sustainable changes	47 (75.8%)
After implementing environmentally sustainable changes	15 (24.2%)

### Anesthesia Providers’ perception of environmental sustainability importance in their Professional Practice

**Fig. 1:**
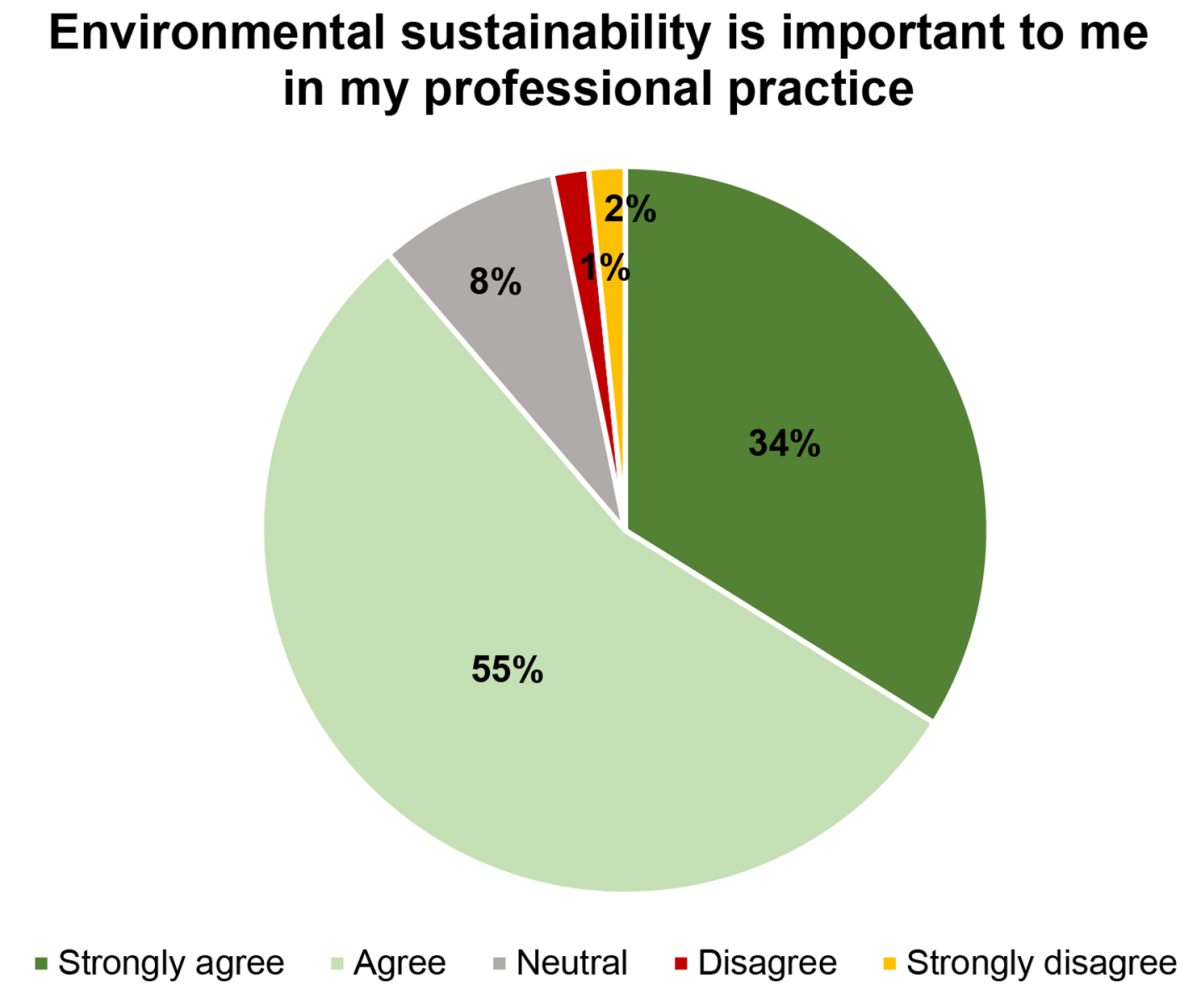
A detailed distribution of responses to the statement: Environmental sustainability is important to me in my professional practice

A total of 55 participants (88.7%) agreed that environmental sustainability is essential in their daily professional practice. Only two participants (3.2%) disagreed with this statement. A detailed distribution of responses to this question is shown in Fig. 1.

### Negative environmental impacts Anesthesia Providers identify in their daily practice

#### Anesthesia-related waste management

Fifty-one out of 62 (82.3%) participants emphasized that one of the significant environmental challenges in anesthesia is the production of a large amount of waste, which is not always properly recycled and should be optimized.

*“We produce a lot of waste: many plastic and metal items are thrown away without being recycled; and excess medicines are carelessly disposed of. Many anesthesia items have to be discarded via special medical waste, which is often not the case.” Participant 31*.

#### Use of disposables

Twenty-eight out of 62 (45.2%) participants underlined their concerns about the use of disposables. Single-use fiberoptic bronchoscopes, laryngoscope blades, Magill forceps, scissors and anesthesia breathing masks have been repeatedly mentioned as critical factors in producing excessive waste.

*“I am concerned about the massive and unfortunately often also not indicated use of disposable products, some of which contain highly complex components that pollute the environment.” Participant 53*.

#### Environmental impact of anesthetics

A total of 27 participants (43.5%) mainly emphasized the ecological damage caused by volatile anesthetics (CO2 emissions, ozone layer depletion) and propofol (water pollution).

*“Inhalational anesthesia is still often performed with excessive flow rate” (Participant 31)*, which *“contributes to intensive CO2 emissions and ozone depletion” (Participant 51)*.

*“Propofol contains phenolic rings, which would have to be burned at around 1000 °C to counteract water toxicity.” Participant 16*.

#### Wasteful electricity consumption

Wasteful electricity consumption was also identified as a significant factor harming the environment (6/62 (9.7%)).

*“Excessive and unnecessary electricity consumption due to many devices that are always on and could be switched off when not in use.” Participant 11*.

### Actions to make daily anesthesia practice more environmentally sustainable

A total of 59 participants (95.2%) stated that they take measures to make their professional practice more sustainable.

#### Environmentally preferable choice of anesthetics

The most frequently reported method of making anesthesia more sustainable was an environmentally preferable choice of anesthetics (29/62 (46.8%)). The participants indicated that they were trying to minimize the use of inhalational drugs and increase intravenous anesthesia if clinically possible. The participants underlined that if inhalation anesthesia is clinically indicated, they try to reduce the climate impact by minimizing the fresh gas flow. The choice to use 1% propofol for shorter anesthesia and 2% propofol for more extended anesthesia also dominated responses.

*“I try to reduce the use of inhaled anesthetics as much as possible” (Participant 36) and, if nevertheless indicated, “conducting inhalation anesthesia with minimal flow” (Participant 30).*

*“Use Propofol 1% for short procedures or do not break an ampoule of 50ml Propofol 2% but use, e.g., 20ml Propofol 1%.” Participant 52*.

#### Conscious planning of anesthetic supplies

Conscious planning of anesthetic supplies, was reported by 25/62 (40.3%) participants. Participants emphasized the need for effective management of anesthetic supplies (medications, airway equipment), including planning in advance and, in such a way, avoiding waste.

*“I try to keep medications and anesthetic materials sterile if possible and unpack them immediately before use to prevent unnecessary waste.” Participant 29*.

#### Recycling and reusing

Eight of 62 respondents (12.9%) identified recycling and reusing as critical measures to make anesthesia practice more sustainable.

*“I reuse as much as possible when feasible and legitimate.” Participant 22*.

#### Encouraging ecological behavior among colleagues

Encouraging and teaching ecological behavior among colleagues was also mentioned as an essential factor in influencing sustainability in anesthesia (3/62 (4.8%)).

*“I try to motivate my colleagues to separate plastic into recycle bags, as well as to conduct inhalation anesthesia with low fresh gas flow.” Participant 29*.

### Barriers that anesthetists face when achieving environmental sustainability in their daily practice

#### Need for knowledge/teaching

The most frequently mentioned barrier when achieving sustainability goals in anesthesia practice was the need for knowledge/teaching in sustainability themes (14/62 (22.6%)).

*“Unbalanced knowledge: advantages and disadvantages of volatile and intravenous anesthetics are often unknown.” Participant 15*.

#### Missing opportunities to implement sustainable practices

Missing opportunities to implement sustainable practices in daily work was emphasized by ten out of 62 (16.1%) participants. The issues predominantly mentioned were: the need for more infrastructure regarding recycling, too many disposables and the feeling of being too small to make a substantial difference.

*“I have no way of influencing waste production at a high level. I can only do it to a small extent.” Participant 10*.

#### Unwillingness to change established norms and adopt new approaches

Seven out of 62 respondents (11.3%) highlighted resistance to changing established norms and adopting new practices as a challenge in achieving sustainability goals.

*“Some colleagues have no interest in these topics and are unwilling to change anything out of convenience with outdated standards.” Participant 31*.

#### Patient safety and hygiene standards

It was also highlighted that hygiene standards adapted to patient safety norms also significantly increase the use of disposable items and, consequently, waste production (7/62 (11.3%)).

*“One of the most significant barriers is the hygiene regulations, which are essential because of patient safety.” Participant 8*.

### Factors leading to non-compliance with internal anesthesia sustainability guidelines

#### Patient-related/medical reasons

The most common theme was patient-related/medical reasons, which was mentioned by 40 out of 62 participants (64.5%).

*“Many of our patients do not fit the norm for intravenous anesthesia due to their age, severe morbidity, or physical or cognitive limitations and require adjusted monitoring. Dogmatic restrictions on using bispectral index neuromonitoring or lack of education may lead to compensatory behavior of either using only sevoflurane or increasing patient safety by using a mixture of sevoflurane and propofol.” Participant 34*.

#### Adherence to established habits

The second frequently mentioned theme was adherence to established habits (12/62 (19.4%)).

*“Routine “tunnel vision” is the problem.” Participant 56*.

The main themes identified in the open-ended questions responses, with the number of participants and percentages, are provided in Table 3.

**Table 3:** The main themes identified in the responses, with the number of participants and percentages

**Negative environmental impacts anesthesia providers identify in their daily practice**	**Measures to make daily anesthesia practice more environmentally sustainable**
• Anesthesia-related waste management *51/62 (82.3%)* • Use of disposables *28/62 (45.2%)* • Environmental impact of anesthetics *27/62 (43.5%)* • Wasteful electricity consumption *6/62 (9.7%)*	• Environmentally preferable choice of anesthetics *29/62 (46.8%)* • Conscious planning of anesthetic supplies *25/62 (40.3%)* • Recycling and reusing *8/62 (12.9%)* • Encouraging ecological behavior among colleges *3/62 (4.8%)*
**Barriers that anesthetists face when achieving environmental sustainability in their daily practice**	**Factors leading to non-compliance with internal anesthesia sustainability guidelines**
• Need for knowledge/teaching *14/62 (22.6%)* • Missing opportunities to implement sustainable practices *10/62 (16.1%)* • Unwillingness to change established norms and adopt new approaches *7/62 (11.3%)* • Patient safety and hygiene standards *7/62 (11.3%)*	• Patient-related/medical reasons *40/62 (64.5%)* • Adherence to established habits *12/62 (19.4%)*

## Discussion

This exploratory qualitative descriptive single-center study sheds light on the current situation of anesthesia providers’ environmental awareness, sustainable anesthesia measures they implement in daily work, their knowledge and needs regarding eco-friendly professional practice. Exploring the perception of professionals and identifying their demands concerning the topic is crucial in achieving sustainability goals at the local- and systems levels.

First, it is important to emphasize that the response rate in the current study was quite low (18.1%), which should be taken into account when interpreting the results. Response rates in similar studies range from 11% [21] to 42% [22]. Non-response [27] and self-selection [28] biases suggest that individuals may be inclined to participate based on personal experience or interest, thus limiting the generalizability of the results.

The principal findings show that the participants are predominantly aware of the importance of practicing sustainable anesthesia; are frustrated with the negative environmental impacts of their professional practice; take actions to make their work more climate-friendly; and recognize barriers to achieving it. Participants also identified factors associated with occasional non-compliance with internal environmentally-sustainable anesthesia guidelines.

Almost 90% of respondents agreed or strongly agreed that sustainability is essential in their daily work, and over 95% of participants indicated that they take specific actions to make anesthesia practice more climate-friendly. Compared to previous studies, which have shown that only about a third of anesthesia providers implement eco-friendly practices [20–22], the encouraging findings of the present study demonstrate a substantial and ever-growing interest and consciousness about the topic. Such a difference could be explained by the newly implemented package of measures to promote green anesthesia enforced at the study center but also with increased awareness about the ecological impact of anesthesia.

Frustration with medical waste management was mentioned by over 80% of participants as one of the significant environmental challenges in anesthesia. This finding underlines the dependence of healthcare on single-use items. Anesthesia-related waste management is also a subject of a global consensus statement from the World Federation of Societies of Anesthesiologists, stating that anesthesia providers should develop and implement an institutionally recognized, regularly monitored “5R” approach to reducing anesthesia waste (medications, equipment, energy and water): reduce > reuse > recycle; rethink, research [19].

Respondents’ most frequently reported sustainability-enhancing method in their daily practice was a conscious choice of anesthetics. The global warming potential of inhaled anesthetics is hundreds of times greater than an equivalent mass of carbon dioxide [32, 33]. In addition, some inhaled anesthetics, particularly nitrous oxide, also contribute to ozone depletion [34, 35]. The production and consumption of mentioned medical gases contribute to total global greenhouse gas emissions ranging from 0.01 to 0.1% [36, 37]. With desflurane and nitrous oxide, equivalent carbon dioxide emissions are approximately 40 times greater than those related to sevoflurane at similar gas flow rates [11, 36]. To reduce the negative environmental impact, desflurane vaporizers have been removed from the study center. New technologies to capture (volatiles) and destroy (nitrous oxide) waste anesthetic gases may be promising [36, 38]. Another essential consideration the participants named is the choice of intravenous instead of inhalation anesthesia when clinically safe. The lifecycle greenhouse gas emissions of intravenous propofol are several times lower than those of inhaled anesthetics [11].

The most frequently mentioned barrier when achieving sustainability goals in anesthesia practice was the need for more teaching in sustainability themes. The World Federation of Societies of Anesthesiologists, in a global consensus statement, advises incorporating environmental sustainability principles within formal anesthesia education and leading ecological sustainability activity within healthcare organizations [19]. In the United Kingdom, for instance, the undergraduate medical curriculum involves the principles of sustainable healthcare [39] and the postgraduate curriculum for anesthetic training [40].

Patient-related/medical reasons were mentioned repeatedly in the responses to the factors behind less environmentally friendly choices in anesthesia practice. Patient safety should not be affected by the implementation of sustainable anesthesia practices. Anesthesia greening could only be implemented on principle “*primum non nocere*” - environmentally preferable medicines, equipment and techniques should only be used when clinically safe to do so [19].

### Limitations

This study has several limitations. First, the study has inherent qualitative research limitations. The qualitative analysis provides a detailed description without attempting to assign frequencies to the characteristics identified in the data – rare and more common phenomena are given the same attention.

Second, this study drew from a small sample of 62 participants; therefore, further research is needed to analyze the topic to a broader extent. Despite our efforts to mitigate these issues, survey results remain susceptible to the effects of non-response [27], self-selection [28], and information bias [29]. If certain individuals choose not to participate in a survey, their absence can result in an incomplete and potentially biased data set. As a result, the findings may not accurately represent the broader population, and this limitation may affect the generalizability of the results. When individuals choose to participate based on their personal experiences, interests, or motivations, the sample may not be representative of the entire target population. Although we have made efforts to create a valid data collection instrument that would result in the best-possible generalizability, information bias is also an influential factor that can affect survey results. Furthermore, our study involved clinicians with demanding clinical responsibilities, which could have resulted in nonparticipation due to time constraints.

Moreover, this is a single-center study performed at a university hospital with the implemented package of measures promoting green anesthesia and a high standard of care in Europe – these implemented practices may have influenced the participants’ opinions on sustainability issues, and anesthesiologists’ perceptions may vary in different settings.

However, given the relatively small sample size and single-center design of the study, awareness within a center with a green anesthesia policy may demonstrate how such initiatives can influence anesthesia providers’ perspectives on sustainability issues and inspire less environmentally conscious institutions to adopt sustainable practices.

## Conclusion

This study showed the growing interest in sustainability themes among anesthesia providers and, consequently, an increasing number of anesthesia team members actively taking measures to reduce their professional carbon footprint at work. Anesthesia providers can implement sustainable changes without negatively influencing their perspective on the issue. In addition, sustainable initiatives have the potential to serve as a motivator and increase their consciousness of this global problem. Nevertheless, there is a need for personal and institutional education about sustainability, which would help to overcome existing barriers to achieving environmental goals. Patient safety always comes first - even though patient-related factors may not always allow the most environmentally friendly anesthesia choice to be made, greener anesthetics should only be used when clinically safe. Building eco-friendly health systems begins with environmental sustainability considerations - at the patient-physician interface, hospitals and their purchase departments, systems and policy levels. One thing is evident by definition - global goals can only be achieved by working together.

### Electronic supplementary material

Below is the link to the electronic supplementary material.

Supplementary Material 1

Supplementary Material 2

Supplementary Material 3

Supplementary Material 4

Supplementary Material 5

## Data Availability

The datasets used and/or analyzed during the current study are available from the corresponding author on reasonable request.

## List of abbreviations

FiO2: fraction of inspired oxygen
SpO2: oxygen saturation
