# Supplementary material for: Environmental sustainability from anesthesia providers’ perspective: a qualitative study
Source: BMC Anesthesiol. 2023 Nov 17;23:377. doi: 10.1186/s12871-023-02344-1 (PMC10655271; doi:10.1186/s12871-023-02344-1)
Supplement: Supplementary file 1 — Supplementary Material 1 [file 12871_2023_2344_MOESM1_ESM.pdf]

Online Survey Invitation

Dear colleagues

Our research team is committed to a consistent and sustainable reduction of CO_2 emissions and minimization of the negative ecological impact of anesthesiological activities.

For this reason, we have created a short survey on ``Ecological Sustainability in Anesthesiology`` to explore your opinion on various aspects of this topic.

Link to the survey (duration max. 8 minutes):
XXX

Your participation would be greatly appreciated and would help to assess and reduce the impact of this issue systematically.

Please do not hesitate to contact us if you have any questions.

Best regards
Your research team

Translated with www.DeepL.com/Translator (free version)
