# Supplementary material for: Environmental sustainability from anesthesia providers’ perspective: a qualitative study
Source: BMC Anesthesiol. 2023 Nov 17;23:377. doi: 10.1186/s12871-023-02344-1 (PMC10655271; doi:10.1186/s12871-023-02344-1)
Supplement: Supplementary file 2 — Supplementary Material 2 [file 12871_2023_2344_MOESM2_ESM.pdf]

Online Survey

1. Environmental sustainability is important to me in my work practice:
    a. Strongly disagree
    b. Disagree
    c. Neither agree or disagree
    d. Agree
    e. Strongly agree
2. What negative factors / impacts on the environment do you see in your professional practice?
    ____
3. Are you taking measures to make your work processes more environmentally friendly? If yes, which ones?
    ____
4. What obstacles do you encounter when it comes to achieving environmental sustainability in your work practice?
    ____
5. What are the reasons for the occasional non-compliance with established guidelines, e.g., use of sevoflurane in situations where, according to internal guidelines, intravenous anesthesia should be used?
    ____
6. Please indicate age:
    ____
7. Please specify gender:
    ____
8. My role:
    a. Nurse anesthetist in training
    b. Certified nurse anesthetist
    c. Resident 1-2 years of training
    d. Resident 3-5 years of training
    e. Resident with > 5 years of experience
    f. Staff anesthesiologist
    g. Senior consultant
9. Work in anesthesia experience in years:
    ____
10. Start of the work at the study center
    a. Before implementing environmentally sustainable changes
    b. After implementing environmentally sustainable changes

Translated with www.DeepL.com/Translator (free version)
