# Supplementary material for: Environmental sustainability from anesthesia providers’ perspective: a qualitative study
Source: BMC Anesthesiol. 2023 Nov 17;23:377. doi: 10.1186/s12871-023-02344-1 (PMC10655271; doi:10.1186/s12871-023-02344-1)
Supplement: Supplementary file 3 — Supplementary Material 3 [file 12871_2023_2344_MOESM3_ESM.pdf]

Complete translated answers to open-ended questions of the online survey

What negative factors / impacts on the environment do you see in your professional practice?

Participant	Answer
1	A lot of plastic and one-way items such as one-time fiber optics.
2	Use of sevoflurane, in some cases wasteful use of resources and materials.
3	Massive waste of paper in an outpatient clinic.
4	Energy consumption, waste production, accumulation of toxic substances, waste of time resources (which could be used elsewhere in a sustainable way).
5	Waste, climate-damaging volatile anesthetics
6	Plastic waste.
7	A lot of plastic and metal are not recycled.
8	One-way items.
9	Waste, energy consumption, water consumption
10	None.
11	Too much plastic and metal waste, sevoflurane, N2O, too much disposables. Too much electricity consumption. Excessive and unnecessary electricity consumption due to many devices that are always on and could be switched off when not in use.
12	A lot of waste, one-way material, ozone layer destruction.
13	Large amounts of waste production. No correct waste segregation. Use of one-way items (e.g., thermal blankets).
14	A lot of packaging and anesthesia gases. You have to be ready for all eventualities, therefore in the patient zone, the material cannot be disinfected and is then thrown away.<br>Gases have been abolished, but there is more need for syringes, which also have packaging.
15	We produce masses of waste that is not recycled.

16	1. Many disposable materials.<br>2. Excessive fresh gas flow during anesthesias with volatile anesthetics.<br>3. Ozone depletion caused by volatile anesthetics.<br>4. Appropriate decontamination of drugs should be considered, e.g., the phenolic rings present in propofol, which remain after metabolization and excretion and cause water pollution. Propofol contains phenolic rings, which would have to be burned at around 1000°C to counteract water toxicity.
17	Much material from packages and leftover medicines in the garbage.
18	A lot of disposables and plastic.
19	Anesthetic gases, much material/waste.
20	Waste (packaging, disposable material), anesthetic gases.
21	Plastic consumption, disposable materials.
22	Extreme amount of waste generation.
23	Garbage anesthesia material; disposable tableware in the recreation room.
24	Much (too much) use of disposable materials, excessive packaging (often with much more plastic than necessary), no segregation of waste.<br>Rapid extension of anesthesia with gas, despite other options.
25	We use many disposable products, which generate a lot of waste.
26	Waste, toxic substances.
27	Extreme accumulation of plastic waste, which is incinerated as contaminated waste.
28	Disposable material; hygiene also causes extreme amount of waste.
29	Waste, plastic, disposable, sevoflurane.
30	Production of unnecessary waste.
31	We produce a lot of waste: many plastic and metal items are thrown away without being recycled; and excess medicines are carelessly disposed of. Many anesthesia items have to be discarded via special medical waste, which is often not the case. Inhalational anesthesia is still often performed with excessive flow rate
32	Much disposable material is used, little possibility of separation for recycling.

	One has to change clothes every time one leaves the OR; the laundry ends up in the laundry bag every time -> far too much OR clothing which has to be washed after only a brief period of use.<br>Disposable tableware like paper cups, soup bowls.<br>The team would like to wear their surgical caps, which can be washed, but this is not permitted by hygiene. Thus one must put on 1x cap.
33	Volatile anesthetics, vast amounts of (disposable) material and packaging, unnecessary power consumption due to many devices that are constantly running.
34	Disposable devices, volatile anesthetics.
35	Plastic consumption, large amount of waste, disposable material, many gloves.
36	Waste with disposable material such as bronchoscopes.
37	Emission of greenhouse gas, very much waste production, very unsustainable work in almost all areas.
38	High material waste. Little consideration of recyclability. "Top-down management." Too little investment in employee training.
39	Generating unnecessary waste.
40	We are heavy polluting.
41	Much material is consumed, and ends up in the garbage in partly big plastic containers.
42	CO2 emissions (from electricity production, production of (disposable) material, combustion of (disposable) material), hole in the ozone layer (inhaled anesthetics).
43	Gas anesthesia: air pollution. Keeping lights on all the time everywhere: electricity wastage. Material packaging: do we really need so much paper and plastic?
44	Disposable material, anesthetic gases.
45	Little economic efficiency and ecological thinking or action.
46	Mountains of garbage and disposable materials.
47	Misallocation of resources (waste etc).
48	Very much material is packed in plastic.<br>Medicines are not disposed of in the proper drop off.

	Plastic cutlery and paper cups in the kitchen.<br>Cloth clamps/scissors/magillary forceps/masks/laryngoscope blades are thrown away.<br>Night shirts are put on briefly for same day surgery patients then put in the wash and postoperatively the patient is given a new shirt.<br>Medicines are sometimes prepared in advance and may be discarded because the surgery is cancelled for various reasons.<br>Glass is often disposed of in the general discard.
49	Inhalatvies, disposable packaging,material.
50	Ozone layer destruction & CO_2 release due to inhalation anesthetics & disposable plastic incineration.
51	Emission of greenhouse gases and other environmentally harmful substances, which contributes to intensive CO_2 emissions and ozone depletion. Use of disposable materials and plastics.
52	Massive, (unfortunately often also not indicated) use of disposable products, which partly contain highly complex parts/components. (e.g. BIS/NIRS sensors, fiber optics, temperature probes, PAH, transducers...). Standard mounting of the straightening of drugs or anesthesia material.
53	I am concerned about the massive and unfortunately often also not indicated use of disposable products, some of which contain highly complex components that pollute the environment.
54	Gas anesthesia, a lot of disposable material, double-packed material.
55	A lot of plastic waste due to disposable waste, environmental pollution due to electricity consumption, environmental pollution due to toxins.
56	Sevoflurane, N_2O, i.e. volatile anesthetics. Plastic waste from sterile material
57	Contribution to the ecological footprint.
58	None.
59	A lot of waste where unnecessary.
60	None.
61	Amount of waste generated (packaging material, etc.)
62	A lot of waste, anesthetic gas.

Are you taking measures to make your work processes more environmentally friendly? If yes, which ones?

Participant	Answer
1	Less anesthesia with sevoflurane.
2	Digitization as far as feasible within our framework.
3	Reduction of material and energy consumption, reduction of (often unjustified) prescriptions: medications, examinations.
4	Do not use desflurane; draw up medication only if needed.
5	Waste prevention.
6	Consciously use as little material as possible, less gas narcoses.
7	Total intravenous anesthesia.
8	Not yet.
9	None.
10	Avoid gas narcosis. Turn off computers and lamps if not in use.
11	Wear gloves only when in contact with body fluids, unpack material (sterile) only or in patient zone when effectively needed. Use inhalation anesthetics only if necessary, minimize FGF and O2 concentration.
12	Low-flow gas anesthesia.
13	Provide material only when needed.
14	Try to recycle as much as possible. Turn off appliances and lights when they are not needed.
15	Consistent minimal flow during anesthesia with anesthetic gases.
16	Try to use surgical clamps again.
17	Do anesthesia without gas if possible. Throw away medications in the blue garbage and not in the general trash.
18	Planning of material/medicine consumption.
19	Sevoflurane: minimal-flow.
20	Economical use of medicines and utensils.
21	Plastic tableware; as little plastic consumption as possible.
22	I reuse as much as possible when feasible and legitimate, avoid anesthetic gas, and use minimal flow during (gas) anesthesia.

23	Avoid disposable products if possible. Lower fresh gas flow with volatile anesthetics.
24	Generate as little waste as possible. Toxic substances in the correct disposal bucket.
25	If possible, refrain from using sevoflurane - within the scope of the appropriate guidelines.
26	Avoid waste where possible.
27	Low flow for gas anesthesia; use disposable articles only if correctly indicated and unpack shortly before use.
28	Produce as little waste as necessary.
29	I try to motivate my colleagues to separate plastic into recycle bags, as well as to conduct inhalation anesthesia with low fresh gas flow. I try to keep medications and anesthetic materials sterile if possible and unpack them immediately before use to prevent unnecessary waste.
30	Meanwhile, we collect plastic, many bring their own water bottles for filling. Conducting inhalation anesthesia with minimal flow
31	Extensive avoidance of gas anesthesia.
32	Minimal-flow anesthesia.
33	Try to save gloves, use minimal flow with inhalation anesthesia.
34	Consistent application of low flow for gas anesthesia if technically possible.
35	Reduce use of inhaled anesthetics where possible; work as efficiently as possible (less consumption of medication/materials).
36	Efficient material management: targeted use of medications. Advance planning for material-intensive procedures. Teaching employees what and how we can make our behavior more environmentally friendly. `I try to reduce the use of inhaled anesthetics as much as possible.
37	Teaching our team what and how we can make our behavior more environmentally friendly.
38	Waste segregation but unfortunately not yet plastic segregation.
39	Saving materials/using them efficiently.
40	Large material expense, disposable material.
41	Save inhalative anesthetics, save material, save electricity.

42	Extinguish light; plan specifically from the beginning what is needed; unfortunately not everything possible.
44	Desflurane has been abolished, minimal-flow gas anesthesia.
43	I consciously question every material I use; and it was very helpful to work in a private clinic for several years, evaluating purchasing, costs and necessity.
44	Forward planning of materials.
45	None.
46	Paper cups label with name. Unfortunately that's it.
47	Avoid inhalation anesthetics; draw up medication only as needed.
48	Low flow, waste segregation, waste avoidance.
49	Reduction of gas anesthesia.
50	Strict indication (e.g. NIRS, PONV prophylaxis, etc.). Rational use e.g. low flow anesthesia, use of propofol 1% for short interventions or no breaking of an ampoule 50ml propofol 2% but e.g. 20ml propofol 1%. Combination of anesthesia procedures. Rob/Neo instead of Bridion (hormone effect, also in the environment).
51	Segregation of waste according to internal guidelines.
52	Total intravenous anesthesia if possible. Use Propofol 1% for short procedures or do not break an ampoule of 50ml Propofol 2% but use, e.g., 20ml Propofol 1%.
53	No, no additional ones (except save sevoflurane).
54	Yes, adjustment of the flow on the ventilator. I recycle waste following internal guidelines.
55	Use gloves only if necessary, correct syringe size, only draw up/straighten/unpack what is needed, sterilely close opened NaCl.
56	Economical use.
57	Comparison packaging material, sustainable redundant processes.
58	Try not to work wastefully.
59	None.

What obstacles do you encounter when it comes to achieving environmental sustainability in your work practice?

Participant	Answer
1	There is no other possibility. For example, multi-way fiberoptic bronchoscopes are available.
2	Clinic management, slow project development, tenacious implementation of ideas.
3	Lack of understanding; lack of logistics (e.g. to separate packaging by type); exaggerated safety philosophies; lack of more sustainable alternatives.
4	Everything is double and triple packed - waste reduction is hardly possible this way.
5	Rigid standards.
6	When asking why it is not recycled comes the answer: "can not"...
7	Hospital specifications (e.g. for material).
8	One of the most significant barriers is the hygiene regulations, which are essential because of patient safety.
9	None.
10	I have no way of influencing waste production at a high level. I can only do it to a small extent.
11	One-way material that is not degradable or only degradable with difficulty.
12	The infrastructure for recycling needs to be improved; in addition, there are many single-use materials, which also cause a lot of waste.`
13	Packaging cannot be disinfected and is thrown away when it has been in the pat zone, with lots of disposable material.
14	Cumbersome work processes to recycle. Colleagues who act thoughtlessly.
15	`Unbalanced knowledge: advantages and disadvantages of volatile and intravenous anesthetics are often unknown.
16	Unclear what impact certain drugs have on the environment (propofol), plastic packaging/packaging could be collected for recycling,
17	As mentioned above, too much plastic.
18	Here at the USZ, there are too few blue garbage cans for waste medication. At my previous hospital, there was one in every room, so I could dispose of

	all medications (e.g. perfusors after discharge) there. Here, everything (including antibiotic residues) simply flies into the general waste.
19	Procurement by hospital, time pressure.
20	Most products are only disposable material.
21	In general, a lot of material resources are needed in medicine. Unfortunately, I have no idea how to circumvent this circumstance.
22	Undifferentiated workflows, for example, 5ml of medication in 10ml syringes, etc.
23	You can not choose the packages and the material used by yourself.
24	Many institutional processes.
25	Hygiene measures, training of others.
26	Material is ordered almost exclusively on the basis of price. Often, glaring quality deficiencies become apparent (e.g., non-sterile gloves, ECG, etc.), which leads to unnecessary additional consumption, among other things. In addition, almost everything is switched to disposable material, including laryngoscope blades, scissors, clamps, fiberoptics, etc.
27	Hygiene, willingness of employees to accept extra work for this purpose.
28	Lack of choice between reusable/disposable materials, awareness/knowledge.
29	Time management, employees were trained differently, e.g. to flood or drive sevoflurane at high flow rates, which is not necessary. Possibly too lazy to divide up medications. What speaks against it is that of course more material and more waste is consumed. Waste separation of cardboard, paper and plastic, the USZ would still have a lot of catching up to do if it is not lived from above and by all then no one will adhere to it.
30	Hygienic guidelines/regulations from the USZ.
31	Some colleagues have no interest in these topics and are unwilling to change anything out of convenience with outdated standards.
32	The resistance to counseling by insufficiently informed care providers makes it difficult to change the situation.
33	A lot of throwing away for hygienic reasons, rules like "never do recap" brings even more waste.

34	Greenwashing: We turn off desflurane and then have nothing more to do. This is intellectual dishonesty. The leadership is making it too easy for itself.
35	External conditions/ specifications (pre-packed sets with too much consumables, pre-discussed gas anesthesia, disposable products where they would not be necessary.<br>Lack of time (the less time, the more wasteful work).<br>Little general understanding.<br>No possibilities (e.g. for recycling).
36	On the one hand, there is a lack of material selection. On the other hand, the impression is of dogmatic handling instead of alternative ways of anesthesia management. It would be desirable to focus more on training and sensitizing care providers. The importance of the "why" must be understood by the anesthetists themselves. This requires a shared responsibility towards people and nature.
37	Outdated structures that cannot be questioned.
38	Hygiene, carelessness.
39	Much material is mandatory and is disposable.
40	Unfortunately, not everyone agrees or thinks about it.
41	Outdated thinking patterns, consulting resistance to improve disposal (red, blue bucket).
42	Too many beginners, who first have to be trained professionally and do not know the material costs, are not yet sensitive enough to this and other topics, such as positive suggestion.
43	Processes too rigid, employees too little informed/sensitized.
44	No waste management officer in the OR.<br>Staff untrained.<br>Much disposable material which ends up in the garbage and is not separated.<br>Lots of elaborate plastic packaging.<br>Waste garbage cans not labeled correctly so staff don't know where to dispose of what.
45	Many disposable materials.
46	Processes are predefined, disposable materials are preferred.
47	Disposable material is cheaper than multiple use with sterilization.

48	Dogmas dictated from above, which make rational use difficult or impossible. As a result, young colleagues receive inadequate training, which leads to SOPs taking precedence over generally accepted guidelines right up to management level.
49	More waste separation would be possible: plastic would have to be disposed of accordingly or recycled properly (possibly by an external company).
50	Processes and material ordering cannot be influenced by me.
51	Plastic waste cannot be avoided due to sterile working.
52	Little, everyone can look individually.
53	Sterile work produces a lot of unnecessary waste.
54	Not everyone pulls in the same direction or does not adhere to it.
55	Specifications from manufacturer.
56	None.
57	Many disposable products that generate a lot of waste.

What are the reasons for the occasional non-compliance with established guidelines, e.g., use of sevoflurane in situations where, according to internal guidelines, intravenous anesthesia should be used?

Participant	Answer
1	Laziness, physical and psychological exhaustion, and long working hours. `It is much easier to turn up a sevoflurane vaporizer than to have to keep drawing up propofol.
2	Sometimes patients do not sleep calmly and deeply with i.v. anesthesia despite accurate preoperative medical history. Using low-flow anesthesia with low MAC and reduced administration of i.v. propofol, a much calmer anesthesia can be achieved.
3	Comfort.
4	Sevoflurane is an easily available agent to deepen anesthesia and save propofol – it leads to faster expansion.
5	See SOP.
6	Sevoflurane is used reactively as soon as the patient awakens intraoperatively instead of waiting for propofol bolus to take effect.
7	Not all special cases are listed in the guideline.
8	The possibilities of use according to the instruction, missing BIS.
9	Patient well-being.
10	Laziness. Turning on the sevoflurane is easier than changing the syringe. As a consultant, it is easier to answer ``yes`` to the question ``may I take propofol`` than ``no``. Because ``no`` requires an<br>Explaination of the situation.
11	Not enough deep anesthesia; increased anesthetics demand.
12	If the patient still does not sleep deep enough with propofol.<br>Minimal flow during long surgical procedures fills the tubes with water, clogs the filter, and damages the respirator.
13	1. Habit;<br>2. Lack of interest in the issue;

	3. In rare cases, the awareness that propofol is also not unproblematic in terms of sustainability (better CO2 balance and no damage to the ozone layer, but not insignificant water toxicity - also due to propofol metabolites).
14	Indications, stress, fast work.
15	Cradiac patients, transplant and I think polytox should also get sevolflurane anesthesia!!! So every animal anesthesia is done inhalatively, I do not understand that you should stop that now in humans. Sevoflurane should be used more often again.
16	Sevoflurane does a perceived more stable anesthesia in otherwise difficult patients.
17	Habit, partly better controllability.
18	Pre-existing patient`s conditions, high propofol consumption.
19	Actually, in my opinion, none.
20	Sorry, but sevoflurane is NOT the problem.
21	Being familiar with, e.g., gas anesthesia, ignorance regarding (renewed) guidelines, order of supervisors.
22	If the patient requires it.
23	Patient safety.
24	Unexpectedly high need for anesthesia intraoperatively where continuation of TIVA is inappropriate for patient safety reasons.
25	Better patient outcome.
26	Awareness/knowledge (too strict guidelines).
27	Polytoxicomania and severely cardiac impaired patients is in my opinion the most common reason to justify the use of sevoflurane.
28	If sevoflurane is used then it has justifiable reasons.
29	Habit, comfort.
30	Comfort, supposed benefit for the patient.
31	Patients in whom sevoflurane would not primarily be "permitted" according to the directive, but clinically assessed benefits e.g. only so sleeps deeply enough.
32	Depth of anesthesia with TCI not sufficient.
33	Multifactorial reasons. Often, anesthesia is administered and prepared differently (general vs. regional anesthesia or sevoflurane anesthesia vs.

	TIVA) and a short-term change is not possible. There is often a lack of understanding/confidence for low- or minimal-flow anesthesia.
34	Many of our patients do not fit the norm for intravenous anesthesia due to their age, severe morbidity, or physical or cognitive limitations and require adjusted monitoring.<br>Dogmatic restrictions on using bispectral index neuromonitoring or lack of education may lead to compensatory behavior of either using only sevoflurane or increasing patient safety by using a mixture of sevoflurane and propofol.
35	Dogmatic restrictions on bispectral neuromonitoring use or lack of training may lead to compensatory behavior of either taking only sevoflurane or increasing patient safety using a mix of sevoflurane/propofol.
36	Clear indication or contraindications.
37	Patient-oriented decision regarding the choice of the most suitable anesthetic agent.
38	Many have less experience in TIVA.
39	Necessity, simplicity.
40	Cardiac patients, pediatric anesthesia.
41	Comfort, very sick patients.
42	High drug demand for deep anesthesia.
43	Medical factors, if the material is simply needed, you cannot do without it. But I also think a lot of it is convenience (discarding medications in douche bottles).
44	Habituation, stubbornness.
45	Deep anesthesia in young agitated people who are not allowed to move or be relaxed.
46	Medications drawn up prematurely and then unnecessarily.
47	Patient-specific, intolerance of TIVA.
48	Anesthesia is easier to perform in combination than with very high running propofol, with corresponding influence on the circulation.
49	Contradiction of internal guidelines to established literature.
50	Patient benefit.

51	Patients are individuals and react differently to anesthetics. Not always predictable whether propofol will be well tolerated and/or sufficient.
52	Sevoflurane use based on indications for sevoflurane use.
53	Patient's clinic. If it is clear from the vital signs that a patient has inadequate anesthesia.
54	Decision of a senior anesthesiologists.
55	It is not relevant to salary, you get no bonus.
56	``Routine ``tunnel vision`` is the problem.
57	Medical reasons.

Translated with www.DeepL.com/Translator (free version)
